# Assessing entomological risk factors for arboviral disease transmission in the French Territory of the Wallis and Futuna Islands

**DOI:** 10.1371/journal.pntd.0008250

**Published:** 2020-05-13

**Authors:** Elodie Calvez, Nicolas Pocquet, Atoloto Malau, Sosiasi Kilama, Alefosio Taugamoa, Didier Labrousse, Philippe Boussès, Anna-Bella Failloux, Myrielle Dupont-Rouzeyrol, Françoise Mathieu-Daudé

**Affiliations:** 1 URE-Dengue et autres Arboviroses, Institut Pasteur de Nouvelle-Calédonie, Réseau International Institut Pasteur, Nouméa, New Caledonia; 2 URE-Entomologie Médicale, Institut Pasteur de Nouvelle-Calédonie, Réseau International Institut Pasteur, Nouméa, New Caledonia; 3 Service de l’Environnement de Wallis et Futuna, Mata’Utu, Uvea, Wallis and Futuna; 4 Service de l’Environnement de Wallis et Futuna, Leava, Futuna, Wallis and Futuna; 5 UMR MIVEGEC Univ Montpellier, IRD, CNRS, Institut de Recherche pour le Développement, Montpellier, France; 6 Arboviruses and Insect Vectors, Institut Pasteur, Paris, France; 7 UMR MIVEGEC Univ Montpellier, IRD, CNRS, Institut de Recherche pour le Développement, Nouméa, New Caledonia; Fundaçao Oswaldo Cruz, BRAZIL

## Abstract

**Background:**

The French overseas Territory of the Wallis and Futuna Islands has been affected by several dengue epidemics. *Aedes polynesiensis* is the main mosquito vector described in this territory. Other *Aedes* species have been reported, but recent entomological data are missing to infer the presence of other potential arbovirus vectors and to assess the entomological risk factors for transmission of arboviral diseases.

**Methodology/ Principal findings:**

An entomological prospective study was conducted on the three main islands of the territory to determine the presence and distribution of *Aedes* spp. Larvae, pupae and adult mosquitoes were collected from 54 sampling points in different environments, with a final sampling of 3747 immature stages and 606 adults. The main identified breeding sites were described. *Ae*. *polynesiensis* was found in every sampled site in peridomestic and wild habitats. *Ae*. *aegypti* was only found on the island of Wallis in peridomestic environments with a limited distribution. Two other *Aedes* species endemic to the Pacific were recorded, *Aedes oceanicus* and *Aedes futunae*. To evaluate the ability of local *Ae*. *polynesiensis* to transmit the chikungunya virus (CHIKV), two field populations were analyzed for vector competence using experimental oral exposure of females to CHIKV and infection, dissemination and transmission assays. Results showed that both populations of *Ae*. *polynesiensis* were competent for CHIKV (30% at 7 days post-infection).

**Conclusions/Significance:**

This study showed the ubiquitous distribution and abundance of *Ae*. *polynesiensis* on the three islands and demonstrated that local populations were able to transmit CHIKV. Combined with the presence and expansion of *Ae*. *aegypti* on the main island of Wallis, these data highlight the risk of transmission of arboviral diseases in the territory of Wallis and Futuna and provide relevant information for entomological surveillance and vector control programs.

## Author summary

The French overseas Territory of the Wallis and Futuna Islands, located in the South Pacific, has been affected by several dengue epidemics, but did not face Zika or chikungunya outbreaks, unlike other neighboring islands. The near-exclusive presence of the *Aedes polynesiensis* mosquito in the islands of Wallis and Futuna confirmed the role played by this mosquito as a vector of dengue fever. A local *Ae*. *polynesiensis* population was recently shown to be able to transmit the Zika virus under experimental conditions, but its susceptibility to the chikungunya virus was still unknown, and recent data on the presence of other potential arbovirus vectors were missing. Therefore, we investigated the entomological risk factors for the transmission of arboviral diseases in the Wallis and Futuna Islands. We reported the occurrence and distribution of different *Aedes* species, especially the abundant presence of *Ae*. *polynesiensis* across the territory and the spread of *Ae*. *aegypti* in the island of Wallis. Our results demonstrated the ability of local *Ae*. *polynesiensis* populations to transmit the chikungunya virus. These findings highlight the risk of arbovirus transmission in the Wallis and Futuna Islands and provide relevant data to guide prevention and vector control strategies in the territory.

## Introduction

Vector-borne diseases (VBD) are widespread in the world, especially in tropical and subtropical regions. We are currently witnessing the emergence or spread of some of them in a context of demographic and societal evolution, as well as environmental and climate changes [1–4]. VBD, especially arboviral diseases, represent an important public health problem in the Pacific Island Countries and Territories (PICTs). Indeed, arbovirus outbreaks are recorded in the South Pacific region since the end of the 19^th^ century [5] and PICTs have been regularly affected by dengue fever epidemics caused by the four dengue virus serotypes [6–8]. An increasing number of outbreaks have affected these islands over the past decade, some due to the emergence or re-emergence of arboviruses in this region [9, 10]. The first cases of autochthonous transmission of the chikungunya virus (CHIKV) were detected in New Caledonia in 2011 [11]. Additional outbreaks were described between 2012 and 2014 in Papua New Guinea, Yap State (Federal States of Micronesia), Tonga, Samoa, American Samoa and Tokelau [12, 13]. In 2013, the Zika virus was detected in French Polynesia and spread very rapidly to other Pacific islands: New Caledonia, Cook Islands, Easter Island, then Tonga, Samoa, Marshall Islands, Fiji, until it reached other parts of the world, Latin America and the Caribbean [9, 14, 15].

The transmission of these arboviruses is ensured by mosquitoes of the genus *Aedes*, subgenus *Stegomyia* [1, 16, 17]. *Ae*. *aegypti* is the major widespread arbovirus vector in the South Pacific region [8]. The invasive species *Ae*. *albopictus* is now established in some Pacific islands like Solomon Islands, with an extended range to Fiji and Tonga [18]. Other endemic species such as *Ae*. *hensilli* or *Ae*. *polynesiensis* are also incriminated vectors for the transmission of arboviruses in some PICTs [19–21].

**Fig 1:**
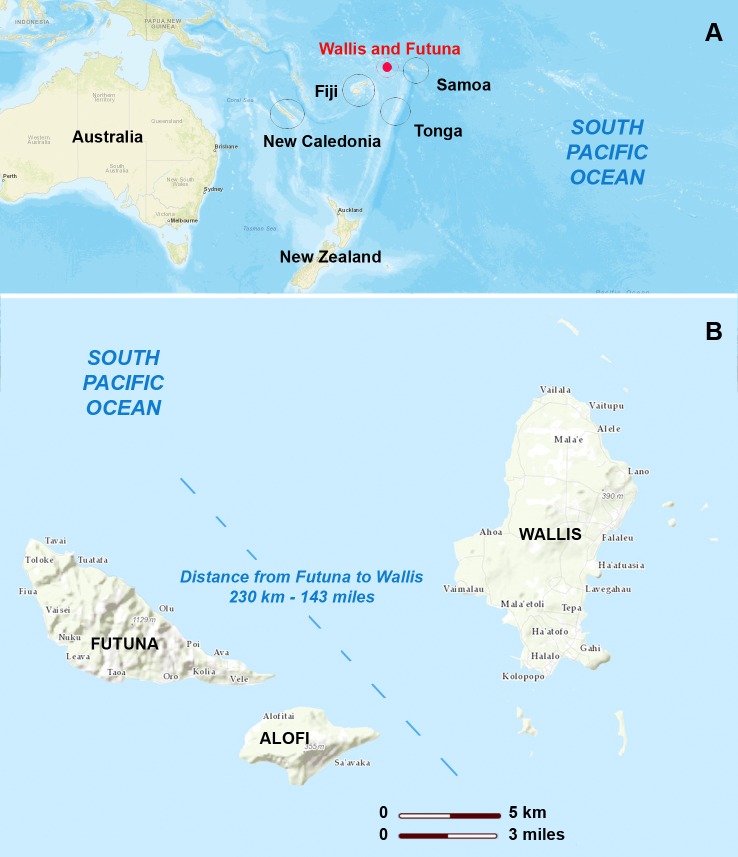
Territory of the Wallis and Futuna Islands. (A) Location of the Territory of the Wallis and Futuna Islands in the South Pacific represented by the red spot. (B) Map of Wallis and Futuna showing the three main islands: Wallis (78 km^2^) to the northeast, Futuna (46 km^2^) and Alofi (18 km^2^) to the southwest. Open source maps from USGS LandsatLook (https://landsatlook.usgs.gov/viewer.html) annotated with Adobe Photoshop 8.0.1.

The Territory of the Wallis and Futuna Islands is a French overseas collectivity in the South Pacific (coordinates 13°18′ South, 176°12′ West) located between Fiji to the Southwest and Samoa to the East (Fig 1A). The territory is made up of two groups of volcanic islands 230 km apart. Wallis Island in the Northeast is composed of a main island and its surrounding lagoon and islets, and the Hoorn Islands in the Southwest are composed of the Futuna and Alofi islands separated by a 2 km wide channel (for convenience these islands will be cited as Wallis, Futuna and Alofi, respectively) (Fig 1B). The total land area is about 142 square kilometers, and the population is about 12,000 inhabitants with almost 70% living on Wallis and the rest of the population on Futuna [22]. The island of Alofi is uninhabited but its lands are used for traditional subsistence agriculture and are thus visited on a regular basis by Futuna inhabitants. The climate of Wallis and Futuna is tropical to equatorial, hot and humid throughout the year, with a warmer and more humid season from November to April.

The Wallis and Futuna collectivity was affected by a large-scale dengue epidemic in 2002–2003 with about 3,000 cases reported in both islands [6, 23]. An entomological survey carried out at that time highlighted the near-exclusive presence of *Ae*. *polynesiensis* on the territory islands. *Ae*. *aegypti* was spotted only once on Wallis Island (in one garden) and was not detected on Futuna [24]. *Ae*. *polynesiensis* is the main vector of lymphatic filariasis in the South Pacific, a parasitic disease caused by the *Wuchereria bancrofti* pathogen, but it is also considered a secondary vector of dengue fever [6, 8, 25]. These observations on Wallis and Futuna confirmed the role played by *Ae*. *polynesiensis* as a dengue vector in these islands. The territory was not affected by dengue epidemics that occurred later in New Caledonia (2008–2009 and 2012–2013), despite several imported cases [26]. Also, it did not experience any autochthonous case of chikungunya or Zika, despite the numerous exchanges with other territories, especially New Caledonia and Fiji which have sustained outbreaks caused by these arboviruses since 2011 [8]. However, Wallis and Futuna recently experienced a dengue outbreak (2017–2018) with 209 autochthonous cases, 197 cases in Wallis and 12 in Futuna (including 8 imported cases from Wallis) [27]. Whether this vector is a less efficient vector than *Ae*. *aegypti* for dengue viruses remains a question, and these observations underline the importance of improving our knowledge on the competence of this mosquito species for arboviruses. A recent experimental work revealed a low vector competence of *Ae*. *polynesiensis* from Wallis for the Zika virus (ZIKV) [28], but the ability of this mosquito to transmit CHIKV in these islands is unknown.

An entomological field survey was conducted in the islands of the territory of Wallis and Futuna in order to characterize and map the different *Aedes* mosquito species, which are confirmed or potential vectors of arboviruses. The objective of the present work was to acquire new entomological data that cover the three islands of Wallis and Futuna to i) check the presence of *Aedes* species previously recorded on the islands, ii) monitor a possible dissemination of these species in the islands and between islands, and iii) control the possible colonization of the territory by new *Aedes* species. Furthermore, we investigated the vector competence of local *Ae*. *polynesiensis* populations for CHIKV to determine the risk of transmission of chikungunya in the territory.

## Methods

### Ethics statement

Animals were housed in the Institut Pasteur animal facilities accredited by the French Ministry of Agriculture for performing experiments on live animals. Work on animals was performed in compliance with the French and European regulations on care and protection of laboratory animals (EC Directive 2010/63, French Law 2013–118, February 6th, 2013). All experiments were approved by the Ethics Committee #89 and registered under the reference APAFIS#6573-201606l412077987 v2.

### Study sites

**Fig 2:**
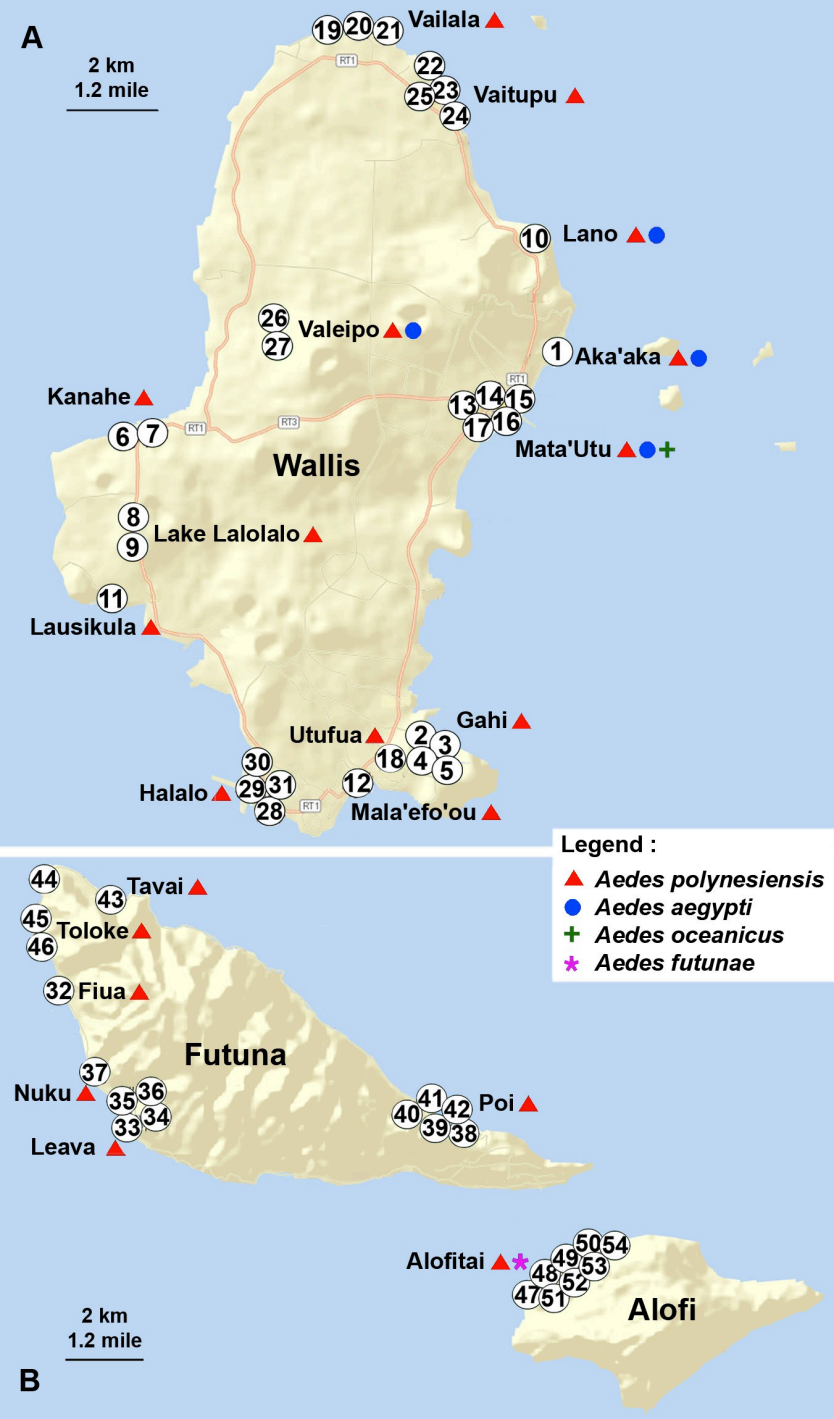
Geographical location of the mosquito sampling sites and distribution of *Aedes* spp on Wallis and Futuna. The numbers represent the 54 sampling points on (A) the island of Wallis, (B) the islands of Futuna and Alofi. The presence of the different *Aedes* species is represented by a sign near the locality name according to the legend on the map. Open source maps from USGS LandsatLook (https://landsatlook.usgs.gov/viewer.html) annotated with Adobe Photoshop 8.0.1.

The three islands of Wallis, Futuna and Alofi were visited for field prospections. The prospection work was conducted during the months of March and April 2016 at the end of the hot and wet season when mosquitoes were the most abundant. As the environment on Wallis and Futuna is of rural and semi-rural type and non-urban, a cross-sectional study was conducted in 20 localities corresponding to villages and wild areas selected to cover the three islands. In these localities, 54 sampling points, at least 100 meters apart, were prospected for immature and adult stages (Fig 2, numbers 1 to 54). These sampling points were selected to represent all the different environments of the islands, from peridomestic to wild habitats.

### Mosquito collection

The survey work focused on *Aedes spp*. but *Culex spp*. mosquitoes were also collected when these two genera shared the same breeding sites or were caught in the same traps. The immature stages of mosquitoes (larvae and pupae) were collected during the survey of breeding sites in the 54 sampling points on the islands of Wallis, Futuna and Alofi. Prospecting sites were either peridomestic, in the immediate vicinity or close to dwellings or places of social life (gardens, churches, *fales-*traditional Polynesian housing with open sides and a pandanus roof), but also wild sites in remote areas (forest). On the island of Alofi, which has no permanent inhabitant, the area chosen for prospection was close to the north-west shoreline of the island, where boats land from Futuna (Alofitai area).

Adult mosquito captures were achieved through BG-Sentinel traps with the BG-Lure olfactory attractant (Biogents, Germany) set up on sheltered terraces for 24 hours. Adult mosquitoes were also captured using a vacuum sucker during day time in peri-domestic places (mainly gardens) and in wilder areas, like in the forests surrounding the Lalolalo lake in Wallis or near the Alofitai beach in Alofi.

### Mosquito identification

Collected individuals, larvae and adults, were identified under a binocular microscope in the laboratories of the *Service Territorial de l’Environnement* in Mata'Utu and Leava localities. Pupae and larvae were kept until emergence to allow identification of adults. Some larvae were conserved in alcohol and later mounted between slides and coverslips for identification under optical microscopes.

### Vector competence

#### Mosquito populations

Two populations of *Ae*. *polynesiensis* from Wallis were studied: WKANA from the locality of Kanahe and WLALO from the Lalolalo lake area. Eggs of the F1 generation were shipped to the Institut Pasteur in Paris, France (Arboviruses and Insect Vectors) to perform the vector competence studies. Emerging adults were maintained at 28°C, 80% humidity with a 16h:8h light:dark cycle and fed *ad libitum* on a 10% sucrose solution. Females were blood-fed several times on anesthetized mice to obtain the F3 generation of mosquitoes used for the infection assays.

#### Viral strain and mosquito oral infections

Five to seven day-old females (F3) were fed with independent infectious blood meals containing CHIKV at a titer of 10^7^ pfu/ml. The CHIKV strain, isolated in 2011 from a patient in New Caledonia (NC/2011-568) [11], belongs to the Asian genotype and harbors an alanine at position 226 in the E1 envelope glycoprotein (GenBank HE806461) [11]. The infectious blood meal was composed of a virus suspension (1:3) and washed rabbit erythrocytes obtained directly from a rabbit (New Zealand white rabbit, Charles River) (2:3) supplemented with 5 mM adenosine triphosphate. The female mosquitoes were allowed to take the infectious blood meal through a capsule (Hemotek system) covered by a pig intestine casing (obtained from a commercially purchased pig intestine) as membrane. After the blood meal, fully engorged females were transferred to new containers and maintained at 28°C, 80% humidity, under a 12h:12h light:dark cycle and fed with a 10% sucrose solution in free access.

#### Infection, dissemination and transmission analyses

For both mosquito populations, 26 to 31 females, for the WLALO and WKANA populations, respectively, were analyzed at 7 days post-infection (dpi) and recorded as infected or non-infected. For each mosquito, different parts of the body were titrated to sequentially determine different rates: abdomen and thorax to determine the infection rate (number of infected bodies/number of mosquitoes tested), the head to determine the dissemination rate (number of infected heads/number of infected bodies), and the saliva to determine the transmission rate (number of infected saliva/number of infected heads). Transmission efficiency was obtained by dividing the number of mosquitoes with infected saliva by the total number of mosquitoes tested, as previously described [28, 29]. For each saliva collection, the females were anesthetized by exposure to cold and their legs and wings were removed. The proboscis was then inserted into an ART filter tip (Molecular BioProducts) containing 5 μl of FBS for a 20 minutes salivation. The body and the head were individually ground in 250 μl of DMEM medium supplemented with 2% FBS. Lysis was carried out during 30 sec at 6,000 rpm and the samples were centrifuged at 10,000 g during 10 min at 4°C. The supernatants were stored at -80°C before analysis. For the viral titration, ground samples serially diluted were inoculated onto Vero E6 cells (African green monkey kidney cell line obtained from the American Type Culture Collection, ATCC CRL-1586) in 96-well plates, incubated for 7 days at 37°C and stained with a solution of crystal violet (0.2% in 10% formaldehyde and 20% ethanol). Presence of viral particles was determined by the presence of cytopathic effect (CPE). Collected salivae were stored at -80°C. For detection and titration of CHIKV, saliva samples were inoculated onto Vero cells in 6-wells plates under an agarose overlay and incubated for 7 days at 37°C. Presence of infectious particles was assessed by the detection of plaques, and titers were expressed as plaque-forming unit (PFU)/mL/saliva.

#### Statistical analysis

Statistical analyses were performed with R v. 3.3.1 [30]. Qualitative variables were compared using Fisher’s exact test, and quantitative variables by a non-parametric test (Wilcoxon test). Statistical differences were considered significant for *p*-values ≤ 0.05.

## Results

### Number and types of breeding sites identified

In the 20 localities and the 54 sampling points prospected on the three islands of Wallis, Futuna and Alofi (Fig 2), 27 capture events of adult mosquitoes were performed and a total of 96 positive breeding sites were identified (Table 1).

**Table 1:** Number of localities prospected, sampling points, adult capture events and breeding sites identified for each of the three islands of the Wallis and Futuna territory.

Island	Number of localities	Number of sampling points	Number of adult capture events	Number of breeding sites
Wallis	13	31	21	56
Futuna	6	15	3	25
Alofi	1	8	3	15
Total	20	54	27	96

The 96 mosquito-producing containers were classified in eight categories (Table 2). 33% of the breeding sites were found in the coconut fruit category. These coconuts were mainly half-shells with extracted pulp, left in gardens or on the beach, but a few of them were entire fruits cracked by rodents. This category was present and predominant on the three islands. Bowls, dishes and boxes were also common breeding sites (18%) present in the three islands and were highly represented in Wallis. Used tires (18%) and buckets and barrels (13%) were also productive containers found on the two inhabited islands of Wallis and Futuna. Some natural breeding sites were found on the island of Alofi, such as natural tree holes and rodent gnawed coconuts, but artificial breeding sites were also found on this island such as a few plastic boxes and half shells of coconuts left by human, or holes made by humans in coconut tree trunks.

**Table 2:** Distribution of the different types of breeding sites sampled on the islands of Wallis, Futuna and Alofi.

		Type of breeding site							
	Number of breeding sites	Coconut fruits and shells*	Bowls, dishes, boxes	Tires*	Buckets, barrels	Plant pots, cuttings	Tree holes	Car wrecks	Boats
Wallis	55	15	13	7	9	8	1	1	1
Futuna	26	9	2	10	3	0	0	2	0
Alofi	15	8	2	0	0	0	5	0	0
Total	96	32	17	17	12	8	6	3	1
%	100	33.3	17.7	17.7	12.5	8.3	6.3	3.1	1

* an isolated coconut shell or used tire, or a pile of any of these items, was counted as a single breeding site.

### Identification of mosquito stages and species collected in the different islands

The collected material was studied for number and distribution of immature stages and adults, and for identification of species (Table 3). Four different *Aedes* species were found on the islands: *Aedes (Stegomyia) polynesiensis* Marks 1951, *Aedes (Stegomyia) aegypti* Linnaeus 1762, *Aedes (Stegomyia) futunae* Belkin 1962, *Aedes (Finlaya) oceanicus* Belkin 1962. The presence of two *Culex* species, *Culex (Culex) quinquefasciatus* Say 1823, and *Culex (Culex) annulirostris* Skuse 1889, was also recorded. The geographical distribution of the four *Aedes* species is indicated in Fig 2.

**Table 3:** Number and distribution of immature stages and adults of the identified *Aedes* and *Culex* species.

	*Aedes* *polynesiensis*			*Aedes* *aegypti*			*Aedes* *futunae*			*Aedes* *oceanicus*			*Culex quinquefasciatus*			*Culex annulirostris*		
	L	P	A	L	P	A	L	P	A	L	P	A	L	P	A	L	P	A
Wallis	1751	279	451	75	15	29	0	0	0	0	0	2	22	0	24	0	0	1
Futuna	790	155	10	0	0	0	0	0	0	0	0	0	157	7	10	4	1	2
Alofi	618	63	114	0	0	0	1	0	0	0	0	0	0	0	0	0	0	0
Total	L + P		A	L + P		A	L + P		A	L + P		A	L + P		A	L + P		A
	3656		575	90		29	1		0	0		2	186		34	5		3
Total	*Aedes spp*.* *:												*Culex spp*.* *:					
	Immature stages Adults			3747 606									Immature stages Adults			191 37		

L: larvae; P: pupae; A: adults

*Ae*. *polynesiensis* was the most sampled species and present in all the 54 sites prospected, with 3,656 immature stages (3,159 larvae and 497 pupae) and 575 adults collected. The species was found in great abundance in wild sites, like the surroundings of the Lalolalo lake in Wallis and along the coast on the island of Alofi.

The *Ae*. *aegypti* species was only found in the island of Wallis, in 5 sampling points corresponding to 4 localities, including Mata’Utu, the most inhabited village of the island (Fig 2). Larvae and adults were collected in the gardens of houses located in the neighborhood of the pier in the locality of Mata'Utu, in Aka’aka, and in piles of tires at the Valeipo garbage dump station. Adult *Ae*. *aegypti* mosquitoes were also captured in a garden in the locality of Lano. A total of 90 immature stages and 29 adults *Ae*. *aegypti* were identified.

The *Ae*. *futunae* native species was only found on the island of Alofi. A larva could be unambiguously identified following observations under microscope. Two adults of the species *Ae*. *oceanicus* were found in the locality of Mata'Utu on the island of Wallis, trapped in BG-sentinel traps. *Culex* species were found only in the islands of Wallis and Futuna, and were not detected in Alofi, with *Cx*. *quinquefasciatus* being much more abundant in the collected samples than *Cx*. *annulirostris*.

Details on the location of the different *Aedes* and *Culex* species collected in the 54 sampling points on the three islands of Wallis and Futuna are provided in S1 Table.

### Vector competence for chikungunya virus

**Fig 3:**
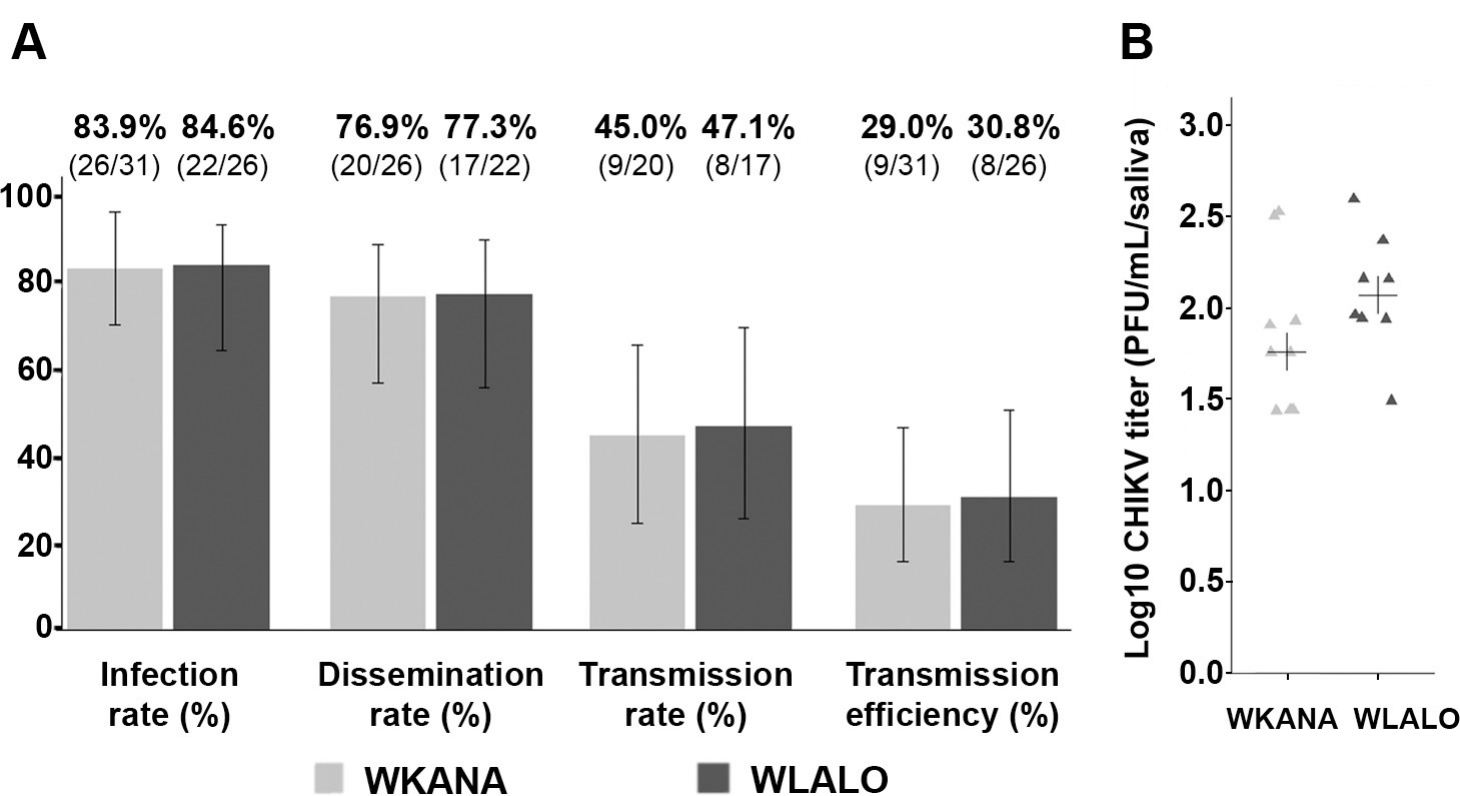
Experimental infections of *Aedes polynesiensis* mosquitoes from Wallis with CHIKV. (A) Infection rate, dissemination rate, transmission rate and transmission efficiency at 7 dpi for the two populations WKANA and WLALO. The percentage value and the number of positive females are indicated above each plot. (B) The titer of infectious saliva is expressed as PFU/mL/saliva for each of the 9 or 8 (for WKANA and WLALO populations, respectively) females whose saliva was tested positive. The cross indicates the median value.

To evaluate the ability of *Ae*. *polynesiensis* from Wallis to transmit CHIKV, we infected two local mosquito populations with a CHIKV strain isolated during the 2011 chikungunya outbreak in New Caledonia. This viral strain was chosen because the Wallis and Futuna population has many exchanges with New Caledonia and therefore the risk of introducing CHIKV from this territory is high. Results showed that, for both studied populations (WKANA and WLALO), more than 80% of female mosquitoes were infected with CHIKV (84% and 85%, respectively), more than 75% of females were carrying viruses in their heads (77% and 77%, respectively) and the transmission rates were about 45% (45% and 47%, respectively). The transmission efficiency was about 30% for both populations, 29% (9/31) for WKANA and 31% (8/26) for WLALO. No significant differences were found between these two populations (Fisher’s exact test, *p >* 0.05) (Fig 3A). We quantified the number of infectious CHIKV particles in the saliva. The viral loads of the two populations were homogeneous, median values of 1.76 and 2.04 PFU/ml/saliva for WKANA and WLALO populations, respectively, and no-significant differences were found (Wilcoxon test, *p >* 0.05) (Fig 3B).

## Discussion

The prospecting work in the three islands of the Wallis and Futuna territory focused on mosquito species that could possibly transmit the main three arboviral diseases (dengue fever, Zika, chikungunya). The work allowed to collect and identify four species of the *Aedes* genus: *Ae*. *polynesiensis*, *Ae*. *aegypti*, *Ae*. *futunae* and *Ae*. *oceanicus*. In peridomestic areas, breeding sites were mostly found in containers used by humans, like coconut shells, bowls, dishes and plastic boxes, used tires, buckets and barrels, etc. Furthermore, breeding sites found in wilder environments also partly resulted from human activities, such as holes in the trunks of coconut palm trees or pierced coconuts. Some of the breeding sites, mainly barrels and used tires, were often highly productive with more than 200 immature stages each. Breeding sites were sometimes shared by two different species of mosquitoes. Associations were *Ae*. *polynesiensis*/*Ae*. *aegypti* in Wallis, *Ae*. *polynesiensis*/*Ae*. *futunae* in Alofi, and *Ae*. *polynesiensis*/*Culex* spp. in Wallis and in Futuna.

*Ae*. *polynesiensis* is the main *Aedes* species on the Territory of the Wallis and Futuna Islands. It was present in all the sites prospected, often in abundance. This species, considered as a secondary vector for dengue viruses is however able to support a dengue epidemic, as shown during the 2002–2003 epidemic recorded in the islands of Wallis and Futuna [31, 32]. The absence of *Ae*. *aegypti* on Futuna confirms the unique role of *Ae*. *polynesiensis* as a vector of dengue in this island (265 cases during the 2002–2003 outbreak and 4 confirmed autochthonous cases in 2018) [23, 27].

*Ae*. *aegypti*, the main vector of arboviruses in the Pacific region, was not found in the islands of Futuna and Alofi. It was however sampled in four sites on Wallis. The presence of *Ae*. *aegypti* on Wallis was already reported during the last two entomological surveys in 2003 and 2007 [24, 31]. At that time, *Ae*. *aegypti* was sampled in a single garden in the vicinity of the wharf of the Mata'Utu locality, and no expansion of *Ae*. *aegypti* distribution was observed in the territory a few years later in 2007 [31]. Our study now shows an expansion of the range of this *Aedes* species to the localities of Aka’aka and Lano to the North (Fig 2). The presence of *Ae*. *aegypti* in used tires at the Valeipo garbage dump station, 5 km apart, might arise from the same initial site or from another source of introduction on the island. Although it is still much less abundant than *Ae*. *polynesiensis*, *Ae*. *aegypti* is therefore well established on Wallis. Entomological monitoring of these sites, with active search for immature stages and adults, and destruction of breeding sites is recommended in order to control the spread of this species to other localities on the island, or to other *Ae*. *aegypti* free islands, like Futuna and Alofi.

Few individuals of two other species of the *Aedes* genus were found on the islands. *Ae*. *oceanicus* was sampled in Wallis and *Ae*. *futunae* in Alofi. Little is known about the role of these two species as vectors of arboviruses or other VBD. *Ae*. *oceanicus* is potentially a secondary vector of lymphatic filariasis, as reported in Tonga [33], or like other species of the subgenus *Finlaya*, such as *Ae*. *(Fin*.*) samoanus* and *Ae*. *(Fin*.*) tutuilae* in other neighboring PICTs (West Samoa and American Samoa) [34–36]. This species was found to play a minor role in the transmission of the Ross River virus in American Samoa in 1979–1980 [34, 35]. It is found in natural habitats, with traditionally reported breeding sites in plant leaf axils such as pandanus and taro. However, immature stages were more recently found in domestic containers suggesting that *Ae*. *oceanicus* has become adapted to these water sources for oviposition [37].

*Ae*. *futunae*, only found on the island of Alofi, is also a species with a natural habitat. Since its description [38], very few references reported this species and its role as a vector is totally unknown [39]. Only one specimen was collected during our survey suggesting a low abundance of this species. However, the prospected site on Alofi, Alofitai, is the place where the inhabitants of Futuna land and embark when visiting Alofi to cultivate their fields. Human presence, even temporary, might be a factor favoring the presence of *Ae*. *polynesiensis* with respect to *Ae*. *futunae*; the abundance of this latter could be slightly greater in parts of the island less frequented by humans. *Ae*. *futunae* was not found on Futuna during our prospection. A larger survey should be performed in the future on the island of Alofi to estimate the population density of *Ae*. *futunae* on this island, and in the uninhabited remote areas of Futuna to ascertain its absence from this island.

*Aedes vexans nocturnus* (Theobald), previously reported in Wallis in 2002 [24], was not found during our survey. The use of BG-Sentinel traps instead of light traps might account for the failure to capture *Ae*. *vexans nocturnus* adult mosquitoes. Indeed, light traps are better suited than BG-Sentinel for the capture of mosquito species displaying nocturnal activity. *Ae*. *vexans nocturnus* specimens were collected using standard New Jersey light traps in Hawaii [40] and CDC-type or EVS-type light traps baited with carbon dioxide in Moorea, French Polynesia, and in Australia [41, 42].

Finally, despite intensive search for both immature and adult stages, and the identification of breeding sites suitable for this species (small peridomestic water-holding containers), the world invasive *Ae*. *albopictus*, now established in various Pacific islands, including the neighboring islands of Fiji [18], was not found during this survey on any of the three islands.

Vector competence of *Ae*. *polynesiensis* from Wallis for CHIKV was investigated. This arbovirus has not been detected so far in the Wallis and Futuna collectivity, with no autochthonous case reported (personal communication from the Wallis & Futuna Health Agency). Our results showed that the *Ae*. *polynesiensis* vector mosquito from Wallis transmitted CHIKV (transmission efficiency of 30% at 7 dpi) under experimental conditions. A vector competence analysis performed on a population of *Ae*. *polynesiensis* from Tahiti (French Polynesia) displayed a weaker transmission of CHIKV (3% at 6 dpi) [43]. These contrasting results may be explained by differences in the experimental protocol of the previous competence work (French Polynesia): mosquitoes of the F14 generation were used for infection; the infection experiments were not conducted with the same CHIKV strain (though it shares 99% homology); and the detection of infectious particles in mosquito saliva was performed on C6/36 cells instead of Vero cells and used indirect immunofluorescent assays. However, in both studies, the vector competence for CHIKV was confirmed. Furthermore, the CHIKV strain that was used in the present study (NC/2011-568) was used in previous studies to evaluate the ability of *Ae*. *aegypti* and *Ae*. *albopictus* from the Americas, and *Ae*. *aegypti* from New Caledonia, to transmit CHIKV [11, 44]. Though results of these studies were heterogeneous, *Ae*. *polynesiensis* appears to be a moderate CHIKV vector compared to some populations of *Ae*. *aegypti* and *Ae*. *albopictus* from New Caledonia and the Americas. More recently, a study carried out under the same laboratory conditions and on the same population of *Ae*. *polynesiensis* from Wallis as in the present work (WLALO) was conducted in order to test the ability of this population to transmit ZIKV [28]. Results of this study showed that less than 5% of the mosquitoes tested transmitted ZIKV. Together, these results suggest that *Ae*. *polynesiensis* from Wallis displays a higher competence for CHIKV than for ZIKAV under experimental conditions in the laboratory.

Our study showed the omnipresence and abundance of *Ae*. *polynesiensis* in the three islands, demonstrating the risk of arbovirus transmission in the territory of Wallis and Futuna, especially for the dengue fever that has already affected the populations on the inhabited islands.

We also showed that local populations of *Ae*. *polynesiensis* are likely to transmit CHIKV. The entire territory of Wallis and Futuna is therefore concerned by the three main arbovirus diseases currently circulating in the Pacific region, namely dengue fever, Zika and chikungunya. Furthermore, the presence of *Ae*. *aegypti* on the island of Wallis, an effective vector of arboviruses, reinforces the epidemic risk of arbovirus transmission on this island.

Although many factors may influence the dynamics of transmission such as vector density, mosquito feeding behavior, vector lifespan, environmental factors [45], there is a high risk for populations in the Wallis and Futuna territory of contracting the two emerging Pacific diseases, chikungunya and Zika, because they are not immune to these viruses.

Surveillance and vector control could be strengthened in this territory. The results of our survey and identification of the different types of breeding sites make it possible to better target vector control according to the *Aedes* species. *Ae*. *polynesiensis* is very opportunistic in finding breeding sites. The natural sites, such as coconuts, tree holes or shattering leaves, represent an obstacle in the control of *Ae*. *polynesiensis* populations. The great number of artificial breeding sites also found in domestic containers left by humans around houses, sometimes very prolific, promotes a high vector density in contact with humans. Measures to manually remove and/or empty these water-holding containers, such as tires, coconut shells, and various other domestic containers, and to protect barrels with mosquito nets, could reduce vector densities in housing vicinities.

Because of its limited distribution and low abundance, preventive measures to control *Ae*. *aegypti* populations should be considered. This control should be achieved through the manual elimination of breeding sites in the different localities where this species was identified, along with localized insecticide treatments.

In addition, other methods such as new vector control strategies using incompatible and/or sterile insect techniques [46–49], may be considered in the future to reduce vector populations in this territory.

Finally, introduction of new species must be monitored. The introduction of *Ae*. *albopictus* from another Pacific island state, such as Fiji which maintains a monthly maritime connection with Wallis and Futuna, represents a threat for the territory. Entomological surveillance at potential introduction points, by air but mainly by sea transport, must be carried out to prevent introduction and establishment of this invasive vector species.

## Supporting information

S1 Table Details on the location of the different *Aedes* and *Culex* species collected in the 54 sampling points on the three islands of the Wallis and Futuna territory. (PDF)
