# Supplementary material for: Assessing entomological risk factors for arboviral disease transmission in the French Territory of the Wallis and Futuna Islands
Source: PLoS Negl Trop Dis. 2020 May 13;14(5):e0008250. doi: 10.1371/journal.pntd.0008250 (PMC7219742; doi:10.1371/journal.pntd.0008250)
Supplement: S1 Table — (PDF) [file pntd.0008250.s001.pdf]

S1 Table. Details on the location of the different *Aedes* and *Culex* species collected in the 54 sampling points on the three islands of the Wallis and Futuna territory.

Island	Locality	Sampling point location*	Sampling point identification	Immature stages in breeding sites**	Adults collection[#]	Other *Aedes* species collected[##]	*Culex* spp collected
**Wallis**	Akaaka	1	WAKAA1	L - P		*Ae. aegypti*	
	Gahi	2	WGAHI1	L - P	Active		
	Gahi	3	WGAHI2	L - P			
	Gahi	4	WGAHI3	L - P			
	Gahi	5	WGAHI4	L - P	Active		
	Kanahe	6	WKANA1		Active		
	Kanahe	7	WKANA2	L - P	Active		*Cx. quinquefasciatus*
	Lake Lalolalo	8	WLALO1		Active		
	Lake Lalolalo	9	WLALO2		Active		
	Lano	10	WLANO1	L	Active	*Ae. aegypti*	
	Lausikula	11	WLAUS1	L			
	Malaefoou	12	WMALA1		Trap		*Cx. quinquefasciatus*
	Mata Utu	13	WMATA1	L	Trap	*Ae. oceanicus*	*Cx. quinquefasciatus*
	Mata Utu	14	WMATA2	L - P		*Ae. aegypti*	
	Mata Utu	15	WMATA4	L - P		*Ae. aegypti*	
	Mata Utu	16	WMATA5	L			
	Mata Utu	17	WMATA3	L			
	Utufua	18	WUTUF1		Trap		*Cx. annulirostris*
	Vailala	19	WVAIL1	L - P			
	Vailala	20	WVAIL2	L - P	Active		
	Vailala	21	WVAIL3	L - P	Active		
	Vaitupu	22	WVAIT1	L	Active		
	Vaitupu	23	WVAIT2	L - P	Active		
	Vaitupu	24	WVAIT3	L - P			
	Vaitupu	25	WVAIT4	L - P	Active		
	Valeipo	26	WVALE1	L - P	Active	*Ae. aegypti*	
	Valeipo	27	WVALE2	L - P	Active		
	Wharf Halalo	28	WHALA1	L - P	Active		
	Wharf Halalo	29	WHALA2	L			
	Wharf Halalo	30	WHALA3	L - P	Active		
	Wharf Halalo	31	WHALA4	L - P			
**Futuna**	Fiua	32	FFIUA1	L - P	Trap		*Cx. quinquefasciatus*
	Leava	33	FLEAV1	L - P			
	Leava	34	FLEAV4	L			
	Leava	35	FLEAV2	L - P			
	Leava	36	FLEAV3	L			
	Nuku	37	FNUKU1		Trap		*Cx. quinquefasciatus* and *Cx. annulirostris*
	Poi	38	FPOI5	L - P			*Cx. annulirostris*
	Poi	39	FPOI3	L - P			
	Poi	40	FPOI1	L - P			*Cx. quinquefasciatus*
	Poi	41	FPOI2	L - P			*Cx. quinquefasciatus*
	Poi	42	FPOI4	L - P			*Cx. quinquefasciatus*
	Tavai	43	FTAVA1	L - P			
	Tavai	44	FTAVA2	L			
	Toloke	45	FTOLO1	L - P			
	Toloke	46	FTOLO2	L			*Cx. annulirostris*
**Alofi**	Alofitai	47	FALOF7	L			
	Alofitai	48	FALOF8	L - P			
	Alofitai	49	FALOF1	L - P			
	Alofitai	50	FALOF2	L - P			
	Alofitai	51	FALOF6	L - P	Active		
	Alofitai	52	FALOF5	L - P			
	Alofitai	53	FALOF4	L	Active	*Ae. futunae*	
	Alofitai	54	FALOF3	L - P	Active		

* Location as indicated on Fig 2 ; ** L : larvae ; P : pupae ; [#] Active : active capture of adult mosquitoes using a vacuum sucker during day time ; Trap : BG-Sentinel trap ; [##] Other *Aedes* species means other than *Aedes polynesiensis,* the latter was found in each of the 54 sampling points.
